# Are Casinos Responsive To Customers Accessing Information about Self-Exclusion?

**DOI:** 10.1007/s10899-025-10442-7

**Published:** 2025-10-23

**Authors:** Margaret Anne Gunnigle, Brianna Morelli, Chance V. Dow, Meredith K. Ginley, James P. Whelan, Rory A. Pfund

**Affiliations:** 1 Tennessee Institute for Gambling Education and Research, Memphis, TN USA; 2 Department of Psychology, Rhodes College, Memphis, TN USA; 3 Department of Psychology, University of Memphis, Memphis, TN USA; 4 Department of Psychology, East Tennessee State University, Johnson, TN USA

**Keywords:** Casino, Self-exclusion, Responsible gambling, Audit methodology

## Abstract

Self-exclusion is a promising intervention for reducing gambling behavior and gambling problems. Casino patrons have reported difficulties when requesting information or exercising the option to self-exclude. Employing an audit methodology, a pair of observational studies explored the accessibility of self-exclusion information from casinos within a mid-size metropolitan gambling market. In study 1, investigators telephoned casino customer service numbers and asked about self-exclusion. In study 2, trained research assistants visited those same casinos to ask multiple staff for self-exclusion information. In both studies, questions were asked about general information about self-exclusion, the physical and online materials on self-exclusion, and directions to the physical and online self-exclusion materials. Phone requests for self-exclusion information were found to be inconsistent and unreliable. Casino staff approached at casinos were generally unable to provide information about self-exclusion beyond references to the physical location of the materials in the casino. Across both studies, casino employees could not reliably provide information about self-exclusion over the phone or in person, revealing potential barriers for individuals who may be interested in pursuing self-exclusion.

**Supplementary Information:**

The online version contains supplementary material available at 10.1007/s10899-025-10442-7.

Casino gambling remains one of the most common forms of gambling. Almost 90% of adults believe that casino gambling is an acceptable form of entertainment, and approximately one-in-four adults engaged in casino gambling in the past year (American Gaming Association, 2023). Despite the expansion of gambling to online platforms like the internet and mobile phones, the revenue generated by land-based gambling has remained stable (*Casino visitation US 2024, *2024).

While not all adults who gamble experience harm, it is estimated that approximately 8.7% engage in risky gambling, and 1.4% experience gambling problems (Tran et al., 2024). Gambling problems negatively impact psychological, financial, and social aspects of not only individuals’ lives, but also their friends and family (Langham et al., 2016). Individuals experiencing gambling problems often encounter stigma from themselves or others, including feelings of shame (Hing et al., 2016). This shame deters help seeking, as secrecy is often used to cope with the stigma (Penfold et al., 2024).

To mitigate the potential for gambling problems at casinos, operators offer self-exclusion programs. Self-exclusion programs are a form of customer protection that allow individuals to voluntarily ban themselves from entering casino properties (Blaszczynski et al., 2007), and these programs are effective in reducing the frequency of gambling and improving psychosocial functioning. The process of self-exclusion varies by jurisdiction, with some jurisdictions mandating licensed operators to offer self-exclusion programs (Kraus et al., 2024), educate customers about the program, and train employees to direct customers to informational materials (LaPlante et al., 2012). Other jurisdictions do not mandate licensed operators to offer self-exclusion programs and instead operators elect to make “reasonable efforts on a facility-by-facility basis to honor any written request, that it not knowingly grant that person access to gambling activities” (American Gaming Association, 2020). Nonetheless, these programs require customers to rely on interactions with venue staff (Pickering et al., 2019), and operators offering self-exclusion should expect their employees to support these programs successfully.

Although self-exclusion programs have been identified as an effective gambling intervention, and operators have made efforts to promote these programs among customers, many barriers to self-exclusion programs have been identified. Individuals have reported that some employees do not know about these programs (Hing et al., 2014; Pickering et al., 2019). Individuals have also reported that they felt anxious while initiating the self-exclusion process and embarrassed during enrollment due to the lack of privacy (Gainsbury, 2014; Hing & Nuske, 2012). Furthermore, individuals have reported that employees struggled to provide compassion and support during the process (Gainsbury, 2014; Hing et al., 2014; Pickering et al., 2019). Further, previous audit studies have revealed that gaming employees often do not assist persons displaying problematic gambling behavior (Hayer et al., 2020; Meyer et al., 2015). These negative experiences may create unnecessary barriers that deter individuals from successfully initiating self-exclusion (Gainsbury, 2014).

Previous research has shown obstacles interfere with the self-exclusion process, yet little is known about deterrents that people considering self-exclusion may experience when seeking information. Public information about self-exclusion is scarce, and those who have self-excluded often learn about the process through counselors (Nelson et al., 2010; Pickering et al., 2019). This lack of engagement suggests that casinos may have shortcomings in communicating self-exclusion policies. Given that self-exclusion is a beneficial tool, the process should be straightforward with minimal barriers. While previous audit studies have revealed that gaming staff do not react proactively to problematic gambling behavior (Meyer et al., 2015; Hayer et al., 2020), the current studies explore what happens when a customer directly asks for information about self-exclusion. To have a comprehensive understanding of further potential barriers, more research outlining the experience of directly gathering information about self-exclusion is needed. The following pair of studies explored the customer experience when requesting information about self-exclusion in land-based casinos.

Using an audit methodology, often referred to as a secret shopper study (Rankin et al., 2022), investigators posed as customers searching for self-exclusion information through telephone and in-person mediums.

In study 1, advertised customer service telephone numbers were called, with the caller requesting information about self-exclusion. Study 2 was executed in person, with investigators asking casino staff about self-exclusion. As self-exclusion programs were legally required for six of the seven casinos at the time of data collection, it was hypothesized that employees would successfully provide the requested information about self-exclusion. The university’s Institutional Review Board determined this study was not human subjects research, as it explored interactions with businesses rather than individuals. The methodology was patterned on previous audit studies (e.g., Meyerson et al., 2024; Uscher-Pines et al., 2023) and guidelines (Rankin et al., 2022). The casinos were not notified of these calls.

## Study 1

### Casino Sample

The casinos selected for this study were near Memphis, Tennessee, which has a market that serves around 1.3 million people (Census profile:Memphis, TN-MS-AR metro area. Census Reporter, 2023). Within this region, there are seven casinos located within a driving distance of about one hour. Six casinos are located in Mississippi, which legally requires casinos to train employees on the self-exclusion process, provide written materials that outline the procedure for self-exclusion, and provide information on where to obtain the form for self-exclusion (*13 Miss. Code. R. 3–10.4.4 - Duties of Casino*, n.d.). The seventh casino is located in Arkansas, which does not have regulation requiring self-exclusion. However, this casino voluntarily provided self-exclusion and stated on their website that information about their program could be accessed by connecting with an employee or security officer in person.

## Method

### Procedure

When calling casino customer service numbers, research assistants (RAs) followed a script that was created to inquire about self-exclusion over the telephone. RAs were trained to complete these calls in a comfortable, relaxed voice and with fidelity to the script. Training occurred through practice phone calls, where RAs role-played “customers” and casino customer service agents. Following the script, RAs requested general information about self-exclusion and any online or physical information (See Supplemental Material 1). General information was defined broadly as employees’ abilities to supply a definition of self-exclusion, a description of what the process entails, and/or any additional details of the policy (e.g., durations of the self-exclusion period, explanation of the prohibited activities during self-exclusion, how self-exclusion is enforced, and consequences of breaching the self-exclusion agreement). Calls were made to casinos during the summer of 2024, during the casino’s web-posted daytime customer service numbers. To assess the consistency of casino customer service information, casinos were called twice by different RAs. Assignments to call customer service numbers were randomized while assuring that no RA called the same casino twice.

Immediately following each call, RAs recorded their experience on a caller experience form. Call duration and time spent on hold were recorded. In addition, RAs recorded what self-exclusion information was received, and they rated their customer service experiences with the casino using four seven-point Likert scale questions. Included were ratings of attentiveness (1 = *very dismissive*, 7 = *very attentive*), demeanor (1 = *very judgmental*, 7 = *very accepting*), friendliness (1 = *very unfriendly*, 7 = *very friendly*), and knowledge of self-exclusion (1 = *very ill-informed*, 7 = *very knowledgeable*). Immediately following the phone call, RAs documented all pertinent customer service experience details.

## Data Analysis Plan

Analyses were conducted using Statistical Package for Social Sciences (SPSS) Version 29 (IBM Corp, 2022). The means and standard deviations were calculated for total time spent on hold and number of employee interactions during the calls. Frequencies were calculated to determine whether casinos provided general information about self-exclusion, the presence and location of on-site information, the presence and location of online information, the directions to both on-site and online information, whether informational material could be mailed, and whether the RAs received material in the mail. The consistency of the information provided across the first and second telephone calls was also calculated.

## Results

### Time Spent Requesting Information

One casino’s web-posted customer service number was incorrect, so 12 total calls were made instead of the planned 14 calls. Of the 12 telephone calls, the average call length was 7 min (*SD* = 4.16; range of 1 min to 15 min), and the average total time on hold was 3 min (*SD* = 4.25; range of 0 min to 14 min). RAs spoke to between one and four employees, with an average of two employees during a call (*SD* = 0.94; See supplemental material).

### Information Provided by Casinos

**Fig. 1:**
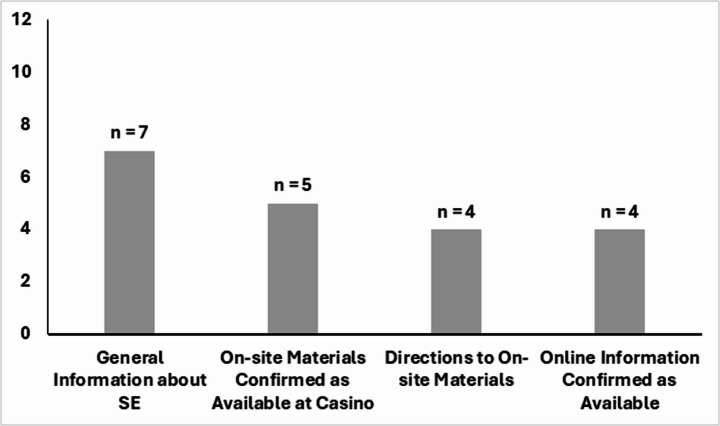
The count of calls (*n* = 12) that resulted in the reception of correct information about self-exclusion. Note. Callers asked for explanations of self-exclusion (e.g., general information), whether on-site or online information was present, and directions to the information

Figure 1 displays the count of casinos that provided the requested information. Across the 12 calls to casinos, seven of customer service operators could provide general information about self-exclusion. The availability of informational materials on site at the casino was confirmed on five calls, and the location of this information within the casino was identified on four calls. When asked about online information, its existence was confirmed during four of the calls, but only three (*n* = 3) provided information about the online location.

### Information Reliability

**Fig. 2:**
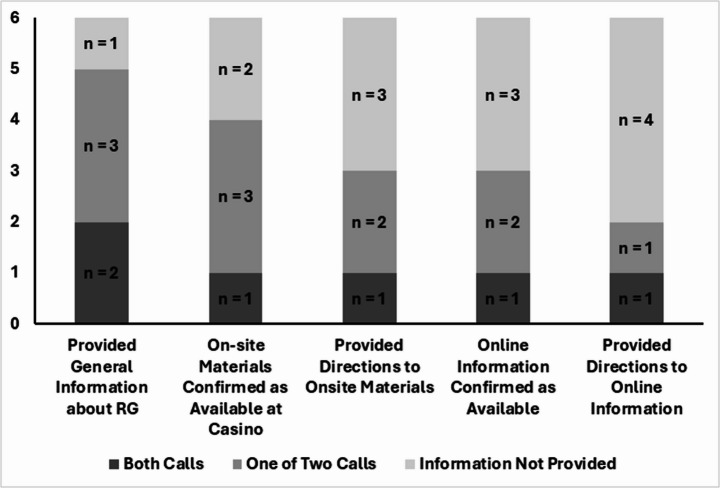
The count of casinos (n = 6) that successfully provided self-exclusion information during one phone call, both phone calls, or neither phone calls. Note: Seven casino properties were called two times, and then were evaluated on the consistency (e.g., the same information) of the provided information. The seventh casino’s web-posted customer service number was incorrect and was never reached

When asked for general information about self-exclusion, only two of the six casinos reliably provided information during both calls to their customer service number (see Fig. 2). Half of the casinos (*n* = 3) provided general information for one, but not both, of the calls. When asked about self-exclusion informational materials at the casino, one of the casinos confirmed the existence of on-site materials during both calls, and half (*n* = 3) confirmed the existence of on-site materials during one of two calls. One-third did not provide information during either call.

### Customer Service Experience

Table 1 displays the customer ratings and recorded experience during each call made to casinos. When assessing whether calls were attentive or dismissive, calls were generally rated as neutral (*M* = 4.7, *SD* = 3.2). Both RAs who called Casino #5 rated calls as somewhat dismissive. In the second call made to Casino #5, the RA reported being hung up on after being placed on hold. In the second call made to Casino #6, the RA rated the call as neutral, reported that it was brief, and that they felt they had to ask questions and press for more information.

**Table 1:** The table below displays the likert scale ratings of employee attention, judgement, friendliness, and knowledge of self-exclusion information. Narrative summaries detailing the customer experience during the first and second calls to each casino property are also displayed

Casino #	Call	Ratings	Summary
Casino #1	1st	Attentive Neutral Neutral Knowledgeable	When I called the number and asked about SE I was quickly transferred to another employee, who then transferred me again. I was on hold for over 5 min waiting for the 3rd employee. However, when I did get to talk to her she seemed knowledgeable on SE and was able to give adequate information. She was very stern on the fact that I would have to come to the casino (or gaming commission) in order to receive the materials.
Casino #1	2nd	Neutral Neutral Neutral Neutral	The first employee told me I had to talk to someone in the casino resort and then asked me if I wanted to self-exclude from the cable gaming or something else (it was a little hard to hear). I said I just wanted general info about self-exclusion, and she transferred me to someone else. This second person didn’t say anything until I spoke first, and the second person said I would need to talk to security, but I was left on hold for 12 min and I ended the call
Casino #2	1st	Somewhat Attentive Neutral Somewhat Friendly Somewhat Ill-Informed	Overall, the experience was short, even when I was placed on hold for a moment. The first woman I spoke to seemed nice but confused when I asked about self-exclusion, so she transferred me to I think the security department. The second guy was also confused, and he told me he’d hand me over to his supervisor, who is the one who gave me the number. This call took about 7–8 min including the times for when I was placed on hold. I think a really interesting thing is that when I asked the guy after he gave me the number if there were materials in the casino, he told me to call the number because everything dealing with self-exclusion is done through the gaming commission in DEIDENTIFIED.
Casino #2	2nd	Somewhat Dismissive Neutral Somewhat Friendly Somewhat Ill-Informed	The woman I talked to seemed very caught off guard when I initially mentioned SE. She told me that everything must be done at the gaming commission, and if I wanted more information I should google it. When asked about any materials within the casino, she said there were flyers hanging up with more information, but she couldn’t tell me what that information was. Overall she was friendly but not very knowledgeable on SE.
Casino #3	1st	Somewhat Attentive Neutral Very Friendly Very Ill-informed	I only got through the first question on the script, and through one follow-up question. After asking about self-exclusion, the employee repeated the words back to me, and then said she was going to “check for information” and “be right back”. I was placed on hold again. When she came back, she apologized for keeping me on hold and said “what is self exclusion?” I said “It’s a casino policy, can you refer me to someone who can help?” She then asked me “is it under responsible gaming?” and I said yes. She told me she would transfer me to the responsible gaming department. I got transferred and just heard this beeping sound for around 15 s, then the call ended. Overall, she was very nice, but did not know anything about self exclusion, and I was never successfully transferred to anyone else.
Casino #3	2nd	Attentive Neutral Very Friendly Neutral	Well, after getting through to the employee, she asked me to repeat the question to her. After that I was put on hold for 1 min, when she came back, she asked if I was still there. As I was responding back with “yes” the call ended. I’m not sure if it was accidental or not.
Casino #4	1st	Attentive Neutral Somewhat Friendly Very Knowledgeable	The employee that gave me the information seemed knowledgeable and was helpful. The most negative takeaway from this experience would be the wait time to actually get a human on the line. After that I had to get transferred twice to get the employee I needed to talk to, but she seemed like she knew what she was talking about.
Casino #4	2nd	Attentive Somewhat Accepting Friendly Knowledgeable	Overall it was a pretty informative experience. I was told what self-exclusion was and the process I need to take. I was told where to find pamphlets and forms within the casino. I was directed to the DEIDENTIFIED gaming commission website. I was told that the SE applies to every casino in DEIDENTIFIED not just DEIDENTIFIED. All employees were nice and receptive to questions.
Casino #5	1st	Somewhat Dismissive Neutral Somewhat Friendly Somewhat Knowledgeable	The overall the conversation was short, most of it I was on hold. I was not sure which button to press and before I could decide the call started ringing but I was immediately on hold. The only information she could provide is that the security guard has the forms, and it is the only place you can do it. When I asked if there was any other information she could suggest, she explicitly said “no”. She did wish me luck before ending the call.
Casino #5	2nd	Somewhat Dismissive Somewhat Judgmental Somewhat Unfriendly Very Ill-Informed	I was placed on hold for like 2 min before speaking to an actual person. While I waited, they played music that sounded like it would come from an ice cream truck. When a real person answered me, the employee sounded confused and told me to “hold on.” The music started again so I thought I would be on hold again for a few more seconds. I was on hold for 4 s before my call ended. At first, I thought maybe I accidentally ended it, but I think the employee hung up on me.
Casino #6	1st	Neutral Neutral Somewhat Friendly Somewhat Knowledgeable	It was a very short conversation, and it seemed like I had to get more information by asking questions. The only information given was to come in and fill out some paperwork, and that the exclusion lasted for two years.
Casino #6	2nd	Somewhat Attentive Somewhat Accepting Friendly Knowledgeable	I was transferred from one security employee to another. When I asked about general info on SE she first asked “are you already self-excluded, or do you want to be?” I said that I just wanted general information, she explained that you can go up to any security guard and ask; they will provide forms for you to fill out. She said that the ban lasts for 2 years and that it cannot be lifted until those 2 years are up. When I asked about materials, she put me on hold to go check and she said that there are no pamphlets, but that the website has a lot of info. She gave me directions on how to get to the responsible gaming page. She also explained that you can fill out the form online and mail it in, but you need to include a copy of your ID. She said that they cannot mail anything. After I asked about any other info she suggested I know, she said no, it is up to the guest to come in and they will be encouraged to self-exclude; all the information is online so if you need more information go online. I felt rushed to go online towards the ends.

When rating calls as either judgmental or accepting, the average rating was neutral (*M* = 4.1, *SD* = 0.5). 75% (*n* = 9) of calls were rated as neutral. The only call that was rated as somewhat accepting was one of the calls to Casino #4, where the RA reported that the employee was very receptive to the questions that were asked.

Generally, calls were rated as somewhat friendly (*M* = 5.2, *SD* = 1.2), with 42% of RAs (*n* = 5) rating the calls as somewhat friendly. Both calls made to Casinos #2, #4, and #6 were rated as somewhat friendly or friendly. The only call that was rated as somewhat unfriendly was the second call made to Casino #5, where the RA was placed on hold. After some time on hold, the call was terminated.

The average employee knowledge rating across calls was neutral (*M* = 4.3, *SD* = 2.0). There were two calls that were rated as somewhat ill-informed. During a call to Casino #2, the RA reported that the first two employees they spoke to sounded confused when asked about self-exclusion. The RA in the other call to Casino #2 was not given a clear explanation and was directed to search for information online instead. Two additional calls were rated as very ill-informed. The first was the call to Casino #5, where the RA reported that the employees did not answer any questions. The second call that was rated as very ill-informed was made to Casino #3, where the employee did not know what self-exclusion was and had to have the RA explain that it was a casino policy. After learning that the policy fell under Responsible Gambling, the call was transferred to the casino’s RG representative and was abruptly ended.

## Study 2

This study explored the experience of accessing self-exclusion information at the casino. Specifically, RAs approached three employees: one who worked on the casino floor, one who did not work on the casino floor, and a security officer. This study implemented an actor-observer arrangement to explore two different perspectives, where the actor engaged with the employee and the observer watched from a moderate distance. It was anticipated that casino floor and security employees would be knowledgeable about self-exclusion processes, while those not on the casino floor might be aware of available information but not the self-exclusion process.

## Method

### Procedure

The script used in Study 1 was adapted to fit an in-person modality. Two RAs, who were over the age of 21, underwent training to communicate with casino employees effectively. The training included practicing the script with other members of the research team until the contents were verbalized in a conversational manner. Once the scripts were successfully recited from memory, casino visits were scheduled. This script contained requests for the following information: general information about self-exclusion, confirmation that this information was available within the casino property and on the casino’s website, and directions to the materials and website content. The full script is in the supplementary materials.

RAs visited each of the seven casinos during the summer of 2024 in the daytime and during the weekend. The sequence of casinos was randomized prior to the visitations. RAs were also randomly assigned to the role of actor or observer via a coin toss before entering the casino property. Then, RAs traveled together to different parts of each casino (e.g., cashier desk, automated teller machines, player services) to verify the existence of informational material about self-exclusion. Informational material was defined as any written material (e.g., pamphlets, booklets) that focused primarily on self-exclusion.

After entering the casino, RAs sought out an employee with the first role within the randomized sequence. RAs then inquired about self-exclusion following the script. Once all questions were asked, the RAs walked away from the employee to record their experiences separately from one another and not in the presence of the employee. RAs recorded whether they received correct directions to the informational material and any reactions to their experience.

RA experience was rated using seven-point Likert scales, including employee attention, (1 = *very dismissive*, 7 = *very attentive*), employee friendliness (1 = *very unfriendly*, 7 = *very friendly*), and knowledge of self-exclusion (1 = *very ill-informed*, 7 = *very knowledgeable*).

### Data Analysis Plan

All analyses were conducted using SPSS version 29. Percentages were calculated to determine whether three different casino employees provided general information, the presence and location of on-site information, the presence and location of online information, the directions to both on-site and online information, whether informational material could be mailed, and whether the RAs received material in the mail. Percentages were calculated separately for the actor and observer. Means and standard deviations were calculated for seven-point Likert scale ratings of the customer experience.

## Results

### Location of Self-Exclusion Information

Informational pamphlets specific to self-exclusion were found in four casinos. Self-exclusion forms were not publicly available in any location. However, four casinos provided self-exclusion forms after interacting with employees who provided the forms. Only three casinos provided both pamphlets. Information was found at the cashier’s desk in four casinos. Two casinos had pamphlets at the player services desk, and three provided them at automated teller machines.

### Information Received: Actor

**Fig. 3:**
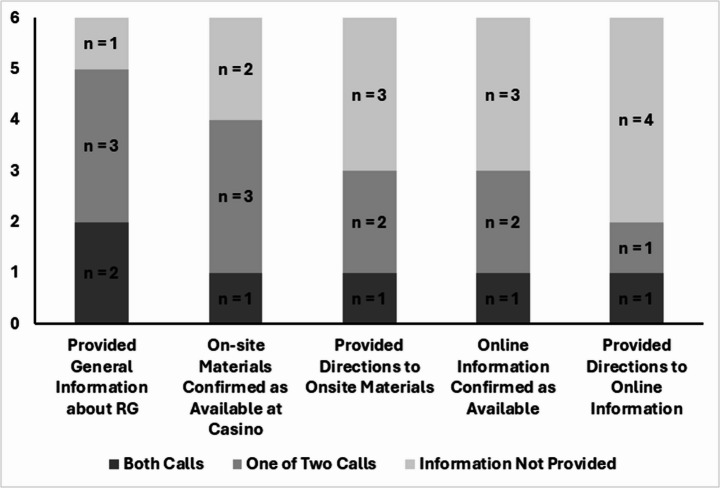
Actor’s experience of whether employees (e.g., on-floor (n = 7), off-floor (n = 7), security (n = 7)) successfully provided self-exclusion information. Note: Actors (e.g., customers who interacted directly with employees) spoke to three different casino employees and requested information about self-exclusion (e.g., general information) and the presence of on-site and online materials

Figure 3 illustrates the percentage of employees in specific roles (i.e., casino floor, *n* = 7, off-floor, *n* = 7, and security, *n* = 7) that were able to provide the requested self-exclusion information. Out of seven possible interactions, general information was provided by 3 casino floor employees. Five out of seven casino floor employees confirmed that informational materials were within the casino, and six provided directions to their location. In four interactions with casino floor employees, RAs were told that there were no materials online. Only one employee could confirm that information could be found online and provide directions as to where it could be found online.

Two of the seven off-floor employees provided general information about self-exclusion. Five confirmed its existence, but only three could provide directions to the location. Five were able to confirm that online information was available, but only two could convey its whereabouts online.

Only one security employee provided information about self-exclusion. Four of seven security employees confirmed the existence of informational materials, but only two provided their location. When asked about the availability of online information, four security employees confirmed that information could be found online but not the location.

### Information Received: Observer

From the observer’s perspective, information was conveyed by four on-floor employees. Six confirmed the existence of on-site material and were able to provide directions to their location. The existence of online information was confirmed by two on-floor employees, and no employee provided directions as to where information could be found online.

General information was received from two off-floor employees. On-site material in the casino was confirmed by six off-floor employees, and directions were provided by two employees. Five off-floor employees confirmed online information, and three provided directions.

Only two security employees could provide general information about self-exclusion. Three security employees were able to confirm the existence of materials and three could provide directions to informational materials. No security employee could confirm the existence of online information, but two provided directions as to their best guess of where information might be located.

### Customer Service Experience

Employee attentiveness was somewhat dismissive (*M* = 3.6, *SD* = 1.7). In 65% of interactions, employees were rated dismissive or somewhat dismissive. One of the RAs who rated Casino #6 as dismissive stated that the on-floor and off-floor employees directed RAs to security for more information, but security gave no information and directed RAs to the off-floor employee. One RA also reported that the off-floor employee from Casino #5 gave short answers and felt they were uninterested in the interactions.

The average employee demeanor rating was neutral (*M* = 4.2, *SD* = 1.2). One RA at Casino #1 recorded that the on-floor employee asked how much money the RA lost and why she needed to self-exclude. RAs rated Casino #7 as somewhat judgmental, as they reported that the security employee kept asking if the RA was trying to self-exclude and if they were going to stop visiting the venue. Both RAs reported leaving the interaction feeling that the security employee was trying to talk the RA out of self-exclusion.

Regarding employee friendliness, RAs rated employees as somewhat friendly (*M* = 4.9, *SD* = 1.1). Employees were rated as somewhat friendly in 29% of RA interactions and were rated as friendly in 36% of RA interactions. RAs reported that the on-floor employee at Casino #2 was very helpful, informing the RAs that they could talk to them for support.

On average, employee knowledge was neutral (*M* = 3.9, *SD* = 1.6), but varied from property to property. RAs reported that employees at Casino #3 were very knowledgeable, as the security employee gave a thorough explanation of the process and provided RAs with forms and pamphlets. RAs reported that on-floor employees at Casino #4 did not know much about self-exclusion and only handed them the informational pamphlets that the casino provided.

## Discussion

These studies investigated the accessibility of self-exclusion information at land-based casinos over the telephone and through in-person visits. In contrast to our hypotheses, information received from employees was often unreliable or incomplete. Requests for information made in person at the casino were more likely to be fulfilled than requests made via customer service calls. However, the fidelity of information provision fell short of what operators promised and regulators required within the jurisdictions of our sample. It was particularly surprising that very few employees were able to verbalize general information about self-exclusion, and, of specific concern, as previous research has highlighted that customers are uneasy and hesitant to seek out this information (Gainsbury, 2014). Unhelpful experiences while seeking out self-exclusion information may exacerbate these hesitations, potentially dissuading individuals from reaching out in the future. Moreover, customers may be hesitant to seek help from casino staff because gambling operators’ offers for help may seem disingenuous (Riley et al., 2018). In the event a person experiences gambling problems and is considering self-exclusion, they would be unlikely to reach out to an operator multiple times to receive information or ensure the veracity of information. As such, it is disappointing that the responses to requests for help were inconsistent and a relatively uninformative experience.

The information received through telephone communications was inconsistent; only one casino provided general information about self-exclusion during both calls. When information was provided, callers were transferred to multiple employees and were placed on hold for long periods of time. When RAs were placed on hold while being transferred to another employee, calls were sometimes abruptly disconnected. Even after speaking with multiple employees, some calls concluded without the RA receiving any information about self-exclusion. Telephone communication has the potential to benefit both customers and operators. Considering that individuals who self-excluded in person have reported embarrassment and privacy concerns during the process (Pickering et al., 2019; Hing & Nuske, 2012), telephone communication offers a discreet and confidential way to access information. As such, contacting casinos using the published customer service telephone line should serve as an easy and accessible medium for customers to learn more about self-exclusion.

Employees also had little knowledge of the self-exclusion process besides where the informational material was located. The lack of self-exclusion usage is attributed to the subpar communication of information that aims to raise consumer awareness of available resources (Gainsbury, 2014; Hing & Nuske, 2012; Pickering et al., 2019). When requesting information about self-exclusion in-person, casino employees were often dismissive. While some employees were attentive and expressed concern, others appeared judgmental, with one even attempting to dissuade the customer from self-excluding. Those seeking out information about the process most likely have experienced gambling problems and are in the process of seeking help, which is an emotionally laborious task (Gainsbury et al., 2014). Additionally, individuals who have experienced gambling problems are often subject to stigmatization from others or themselves (Penfold et al., 2024). Anticipation of this stigma is a significant barrier for seeking help and resources (Penfold et al., 2024).

In the current study, employees appeared insufficiently prepared to offer support or effectively disseminate information. An individual’s experience of requesting self-exclusion information is largely impacted by employee knowledge, which can be improved through training (Pickering et al., 2019). Employee training is important in preparing employees to effectively relay information to customers who may need more information when considering self-exclusion as a viable intervention. Individuals have expressed that their largest barrier to self-exclusion was a lack of knowledge about the program (Hing & Nuske, 2012; Pickering et al., 2019). Self-exclusion has been shown to be a helpful tool for reducing gambling problems (Hing et al., 2015), and casinos who implement these programs with the bare minimum of care are doing a disservice to their patrons. As required by law and the commitments of AGA members, casinos are required to make information about responsible gambling and where to find assistance available to customers (American Gaming Association, 2020, Mississippi Gaming Control Act, 1990, 2020). While self-exclusion can be an impactful intervention for gambling problems, the lack of adequate training may insufficiently prepare employees to provide customer support during the self-exclusion process.

There are other implications for casino operators that require strategies to optimize the customer experience of seeking self-exclusion information. For example, all casino customer service agents can have efficient and cost-effective access to self-exclusion information eliminating the need to transfer calls or put customers on hold in search. A strategy for the casino itself is to designate employees to help with self-exclusion. While casinos implement annual employee training in responsible gambling, it may be unrealistic to expect all employees to hold this responsibility for what is likely to be an infrequent request for help. It might also be best for this responsibility to not be assigned to casino security staff. These teams are primarily tasked with deterring criminal activity and maintaining surveillance systems (Boss & Zajic, 2010). The process of initiating self-exclusion has been found to be stressful (Hing & Nuske, 2012), and approaching security may feel intimidating to customers seeking help. In Massachusetts, for example, external third-party employees are physically present to assist customers with questions including self-exclusion (Louderback et al., 2022). The integration of such player-facing employees has been associated with perceived effectiveness of responsible gambling programs among customers and casino employees (Abarbanel et al., 2022; Beckett et al., 2020).

Another strategy is tasking operators and regulatory bodies with developing systems that monitor compliance with regulations and laws. The audit methodology offers a practical and replicable framework for such oversight. By mimicking the approach of this research, operators or regulators could systematically assess how well staff understand and implement self-exclusion procedures in real-world scenarios. Regular implementation could help identify weaknesses in staff training, policy implementation, and customer interactions that might otherwise go unreported.

Several limitations need to be noted. First, the sample size of the casinos was small, limiting our ability to compare casino properties or regulatory differences. While the study only captures the experience within a singular market, most casino markets within the United States are of modest size, and the casinos in these markets are owned by larger corporations (American Gaming Association, 2023). Second, whether the results were due to gaps in training specific to self-exclusion or customer service training is unknown. The methods of our study did not isolate the two, so it is difficult to conclude whether the results indicate deficiencies in general customer service training or self-exclusion training. Another limitation may be the demographics of the RAs who visited casinos in-person as they were female young adults.

## Conclusion

The findings indicated that licensed casino operators did not fulfill their legal or social responsibility to adequately communicate self-exclusion information to customers. Self-exclusion can be a helpful tool for providing an external barrier to accessing land-based gambling facilities and for preventing further gambling harm (Shaffer et al., 2019). However, in addition to the difficulties experienced during the process of self-exclusion, there seem to be notable barriers to obtaining more information, either in-person or online, potentially deterring individuals considering self-exclusion from taking further action.

## Supplementary Information

Below is the link to the electronic supplementary material.

Supplementary Material 1 (DOCX 19.1 KB)

## Data Availability

Data will be made available upon reasonable request.
