# Supplementary material for: Are Casinos Responsive To Customers Accessing Information about Self-Exclusion?
Source: J Gambl Stud. 2025 Oct 23;41(4):1615–30. doi: 10.1007/s10899-025-10442-7 (PMC12657576; doi:10.1007/s10899-025-10442-7)
Supplement: Supplementary file 1 — Supplementary Material 1 (DOCX 19.1 KB) [file 10899_2025_10442_MOESM1_ESM.docx]

**Supplemental Materials for**

**Are Casinos Responsive to Customers Accessing Information about Self-Exclusion?**

Supplemental Material 1.

*Telephone Call and In-Person Script*

1. “Hi, can you give me general information about self-exclusion?”
2. “Are there any materials in the casino?”

2a. “Where can I find it in the casino?”

1. “Are there any materials online?”

3a. “Where can I find it online?”

1. “Can you mail me any of the materials?”

4a. If yes, provide address from list

1. “What other information can you suggest for me?”
2. “Thank you for your time.”

If asked “what is self-exclusion" or to define self-exclusion:

respond with “it's a casino policy, can you refer me to someone who can help?”

Supplemental Table 2.

*Call information from the first and second call to each of the six casino properties.*

| Casino | Call Number | Call Duration | Time on Hold | Employee |
| --- | --- | --- | --- | --- |
|  |  | Min. | Min. | *n* |
| 1 | 1^st^ | 7.55 | 6.05 | 3 |
| 1 | 2^nd^ | 15 | 13.68 | 2 |
| 2 | 1^st^ | 8 | 1.82 | 3 |
| 2 | 2^nd^ | 2.08 | 0.27 | 1 |
| 3 | 1^st^ | 3.43 | 1.08 | 1 |
| 3 | 2^nd^ | 3.26 | 2 | 1 |
| 4 | 1^st^ | 12 | 9.12 | 3 |
| 4 | 2^nd^ | 7.52 | 0.49 | 3 |
| 5 | 1^st^ | 1 | 0 | 1 |
| 5 | 2^nd^ | 4.54 | 1.33 | 2 |
| 6 | 1^st^ | 9.41 | 0 | 1 |
| 6 | 2^nd^ | 5 | 2.12 | 1 |
|  |  | *M(SD)* | *M(SD)* | *M(SD)* |
|  | Total *n* = 12 | 6.57(4.16) | 3.21(4.25) | 1.83(0.94) |

*Note:* Call number refers to the 1^st^ or 2^nd^ time a casino was called. Call duration refers to the total length of time spent on the telephone. Time on hold refers to the amount of time customers were placed on hold after speaking with a customer representative. Employee number refers to how many different employees were spoken to during the telephone call.
